# ADDRESSING THE LONG-TERM CARE POLICIES IN HAWAII: AN APPLICATION OF BARDACH’S MODEL

**DOI:** 10.1093/geroni/igae098.4307

**Published:** 2024-12-31

**Authors:** Mohammad Hossain

**Affiliations:** Grand Valley State University, Walker, Michigan, United States

## Abstract

In this policy analysis paper, I utilized Bardach’s (1977) model to evaluate three alternative policy options addressing the crucial needs of long-term care in Hawaii. The policy options included the Long-Term Care Insurance (LTCI), the Kupuna Caregivers Program (KCP), and the Long-Term Care Insurance Partnership Program (LTCIPP). These policies were analyzed based on Bardach’s (1977) eight-step model. Further, the evaluation justified the selected policies against several criteria such as financial feasibility, effectiveness, political acceptability, policy sustainability, an overall impact to support older adults towards age in place, effects on employers, and sustainability. The findings of this policy analysis included that the LTCIPP, as a 3rd policy alternative in the list, has more potential to address the service gaps of LTC in the long run compared to the remaining policies. Though the LTCIPP has been progressively growing as a promising LTC initiative, and currently, most U.S. states are entitled to this program, it might not be affordable for potential care recipients from marginal communities such as low-income, homeless, Indigenous communities, etc. Further, this paper acknowledges that addressing the needs for long-term care necessitates comprehensive efforts. Aside from the enrollment in LTCIPP for Hawaii, this analysis recommends promoting and advocating for appropriate funding for the Kupuna Caregiver Program, which is unique even in the U.S., to support low-income families and their care-needed elderly members. The paper suggests that partnering with private entities enhances the government’s chances of avoiding cost-shifting challenges.

